# Evaluation of Antifungal Phenolics from *Helianthus tuberosus* L. Leaves against *Phytophthora capsici* Leonian by Chemometric Analysis

**DOI:** 10.3390/molecules24234300

**Published:** 2019-11-25

**Authors:** Fu-Jia Chen, Xiao-Hua Long, En-Zhong Li

**Affiliations:** 1 School of Biotechnology and Food Engineering, Huanghuai University, Zhumadian 463000, China; shenfujiawlqs@163.com; 2 Key Laboratory of Marine Biology Jiangsu Province, College of Resources and Environmental Sciences, Nanjing Agricultural University, Nanjing 210095, China; longxiaohua@njau.edu.cn

**Keywords:** *Helianthus tuberosus*, antifungal activity, phenolic acids, chemometric analysis, PLS-DA

## Abstract

*Phytophthora capsici* Leonian causes destructive economical losses in pepper production, and a promising source of natural fungicides- *Helianthus tuberosus* leaves was reported. The antifungal activities of different extracts and compounds from *H. tuberosus* leaves against the phytopathogen, *P. capsici* Leonian, were examined by chemometric analysis, including HPLC-MS/MS and multivariate data analyses. Principal component analysis and orthogonal partial least squares-discriminate analysis were applied to examine the four groups of *H. tuberosus* leaves samples, including crude extracts obtained by different methods, including refluxing, macerating, and refluxing under vacuum; four fractions, namely, petroleum ether (PE), chloroform (Chl), ethyl acetate (EA), and n-butanol (NB) fractions; the samples of three *H. tuberosus* cultivars; and the samples at three growth stages of cultivar Nan Yu. The phenolics contents were categorized based on 3,5-Dicaffeoylquinic acid (3,5-DiCQA), 1,5-Dicaffeoylquinic acid (1,5-DiCQA), 3-*O*-Caffeoylquinic acid (3-CQA), and 4,5-Dicaffeoylquinic acid (4,5-DiCQA), which were predominant in all the samples. Antifungal activity assay revealed that Chl and NB fractions were more active against *P. capsici* Leonian with lower IC_50_(half of maximal inhibitory concentration) values, whereas partial least squares-discriminate analysis suggested caffeoylquinic acid isomer(4-CQA), methyl-quercetin glycoside(MQG), and caffeic acid(CA) might be the main active components in *H. tuberosus* leaves against *P. capsici* Leonian. Furthermore, microscopic evaluation demonstrated structural deformities in *P. capsici* Leonian treated with Chl and NB fractions, indicating the antifungal effects of *H. tuberosus* leaves. These results imply that *H. tuberosus* leaves with a high concentration of phenolics might be a promising source of natural fungicides.

## 1. Introduction

*Phytophthora capsici* Leonian is a ubiquitous soil-borne fungus, which can grow in all parts of pepper plants (i.e., fruit, leaf, stem, and root) [1] causing Phytophthora blight of pepper. In China, this phytopathogenic fungus causes destructive economical losses in pepper production. Currently, the major methods employed for pepper blight prevention are cultivation measures and use of fungicides [2], including synthetic, botanical, and microbial fungicides. However, owing to their adverse environmental effects and low effectiveness due to the onset of fungicide resistance, the use of some synthetic fungicides has been banned [3]. Therefore, there is a need for development of new botanical fungicides that can be applied to control phytopathogenic fungal infections, with increasing focus on substances that are selective against these fungi, without being toxic to the ecosystem or humans. The massive structural diversity and versatility of natural components found in plants provide valuable opportunities for developing new fungicides. A variety of spices and herb extracts are being used in food preservation, plant diseases control, as well as medicines.

*Helianthus tuberosus* L. (Asteraceae), also known as Jerusalem artichoke, is widely applied in medicine owing to its pharmacological activities, such as analgesic, antipyretic, anti-inflammatory, antispasmodic, aperient, cholagogue, diuretic, spermatogenic, stomachic, and tonic effects [4,5]. The active compounds in *H. tuberosus* which produce these effects include coumarins, unsaturated fatty acids, polyacetylenic derivatives, phenolic compounds, sesquiterpenes, etc. [6]. Phenolics, predominantly flavonoids, are bioactive compounds with possible antifungal properties on phytopathogenic fungi [7]. Previous studies have indicated that crude extracts (CEs) of *H. tuberosus* leaves and the main phenolics [including caffeic acid (CA), etc.] possess antifungal activities against phytopathogenic fungi such as *P. capsici* Leonian and *Rhizoctonia cerealis* [4]. A structural morphological study of phytopathogens after treatment with antifungal agents could be crucial in elucidating the mechanism of action of these agents [8]. However, to the best of the authors’ knowledge, the mechanism of the components of *H. tuberosus* leaves against *P. capsici* Leonian has not yet been studied. 

Therefore, the aims of this study were to examine the antifungal activities of different extracts and compounds from *H. tuberosus* leaves against the phytopathogen *P. capsici* Leonian through chemometric analysis, including HPLC-MS/MS and multivariate data analyses. It must be noted that multivariate data analysis, including principal component analysis (PCA), orthogonal partial least squares-discriminate analysis (OPLS-DA), hierarchical cluster analysis (HCA) and partial least squares-discriminate analysis (PLS-DA), etc., has been utilized for the identification of bioactive metabolites in natural products [9]. In the present study, PCA, OPLS-DA and HCA were used for distinguishing different extracts and phenolics from *H. tuberosus* leaves, and PLS-DA was performed for modeling the relationship of phenolics and their antifungal activities (represented as half of maximal inhibitory concentration, IC_50_). Furthermore, microscopic evaluation of *P. capsici* Leonian treated with extracts of *H. tuberosus* leaves demonstrated structural deformities in the fungus, indicating the antifungal effects of *H. tuberosus* leaves. 

## 2. Results

### 2.1. Analysis of Major Components in H. tuberosus Leaves

**Figure 1:**
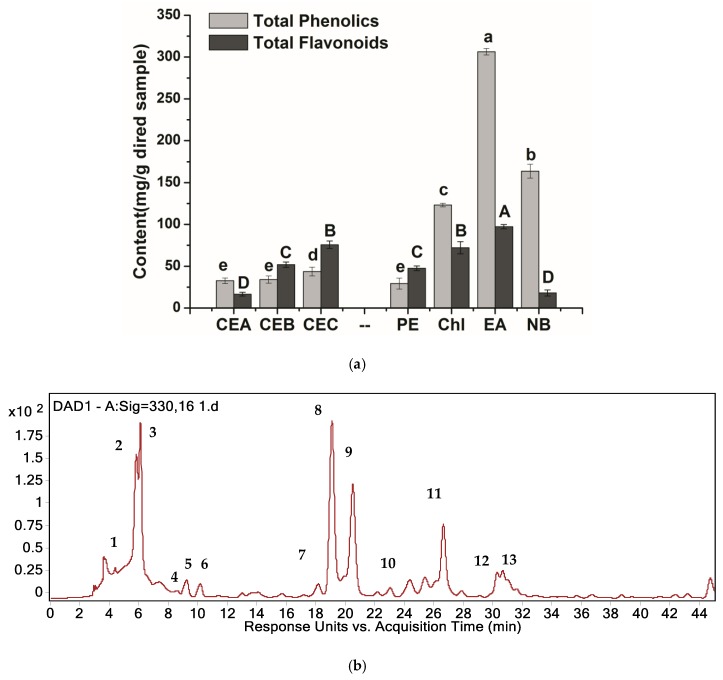
(**a**) total phenolics content (TPC) and total flavonoids content (TFC) in different crude extracts (CEs) (obtained by various extraction methodsand fractions of *H. tuberosus* leaves, mg/g dried sample). (**b**) HPLC-MS/MS chromatogram of CE of *H. tuberosus* leaves. Values are expressed as mean ± SD of triplicate measurements, different letters are significantly different at *p* ≤ 0.05. CE, Crude extract; A, Refluxing; B, Macerating; C, Refluxing under vacuum; PE, CE of Petroleum ether fraction; Chl, CE of Chloroform fraction; EA, CE of Ethyl acetate fraction; NB, CE of n-Butanol fraction. Peak 1: Caffeoylquinic acid isomer, 5-CQA; Peak 2: 3-O-Caffeoylquinic acid, 3-CQA; Peak 3: Caffeoylquinic acid isomer, 4-CQA; Peak 4: Caffeic acid, CA; Peak 5: p-Coumaroyl-quinic acid, PCQA; Peak 6: Feruloyl-quinic acid, FQA; Peak 7: 3,4-Dicaffeoylquinic acid, 3,4-DiCQA; Peak 8: 3,5-Dicaffeoylquinic acid, 3,5-DiCQA; Peak 9: 1,5-Dicaffeoylquinic acid, 1,5-DiCQA; Peak 10: Methyl-quercetin glycoside, MQG; Peak 11: 4,5-Dicaffeoylquinic acid, 4,5-DiCQA; Peak 12: Methoxy-kaempferol glucoside, MKG; Peak 13: Kaempferol-3-o-glucoside, KG.

It has been reported that highly bioactive components occur in the medium and intensive polar fractions [CE of chloroform (Chl), CE of ethyl acetate (EA), and CE of n-butanol (NB)] [4], whereas less effective compounds can be detected in the nonpolar fraction, such as CE of petroleum ether (PE) fraction. In the present study, compounds with antifungal activities, including total phenolics content (TPC), total flavonoids content (TFC) (Figure 1a), and the main phenolic acids, in *H. tuberosus* leaves were qualitatively analyzed (Table 1). In our previous study, HPLC-MS/MS was used to identify the main phenolic acids [5]. Based on their contents in the extracts and fractions of *H. tuberosus* leaves, the main phenolic acids were considered to play dominant roles in antifungal activities (Figure 1b). 

The components of the CE obtained using three different methods, including refluxing (A), macerating (B), and refluxing under vacuum (C), varied. For instance, the TPC in CEC (CE of method C) was higher (43.66 mg/g), when compared with that in the other CEs. With regard to TPC in different fractions, the TPC was significantly higher in the EA and NB fractions (306.38 and 163.73 mg/g, respectively), when compared with those in the PE and Chl fractions. These results demonstrated that the active phenolics in *H. tuberosus* leaves might possess stronger polarity. The TFC in CEC was up to 75.69 mg/g, which was significantly higher than that in the other two CEs. In contrast, the TFC in CEA was as low as 16.55 mg/g, which could be attributed to the high extraction temperature that could have damaged the flavonoids structures [10]. With respect to the TFC in different fractions, the EA fraction presented the highest TFC (97.33 mg/g), followed by Chl fraction (72.15 mg/g), whereas the PE and NB fractions exhibited relatively low TFC. These findings indicated that the active flavonoids in *H. tuberosus* leaves might possess medium polarity.

Without the stringent restriction of all the reference standards, 13 kinds of phenolics were semi-quantitatively detected in *H. tuberosus* leaves based on relative percentage of peak area [11,12,13]. The main phenolic acids in different samples of *H. tuberosus* leaves, including the three CEs obtained by different methods, four fractions, three *H. tuberosus* cultivars, and three growth stages of cultivar Nan Yu, were 3-*O*-caffeoylquinic acid (3-CQA; peak 2), 3,5-dicaffeoylquinic acid (3,5-DiCQA; peak 8), and 1,5-dicaffeoylquinic acid (1,5-DiCQA; peak 9) (Table 1). 

### 2.2. Chemometric Determination of Phenolic Acids 

**Figure 2:**
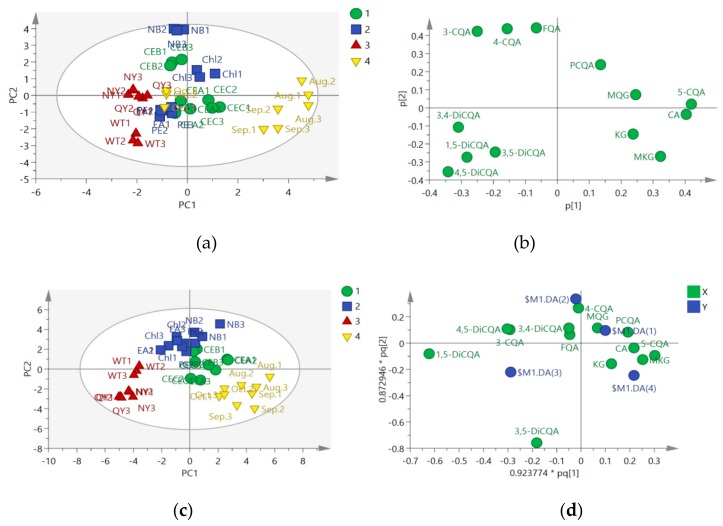

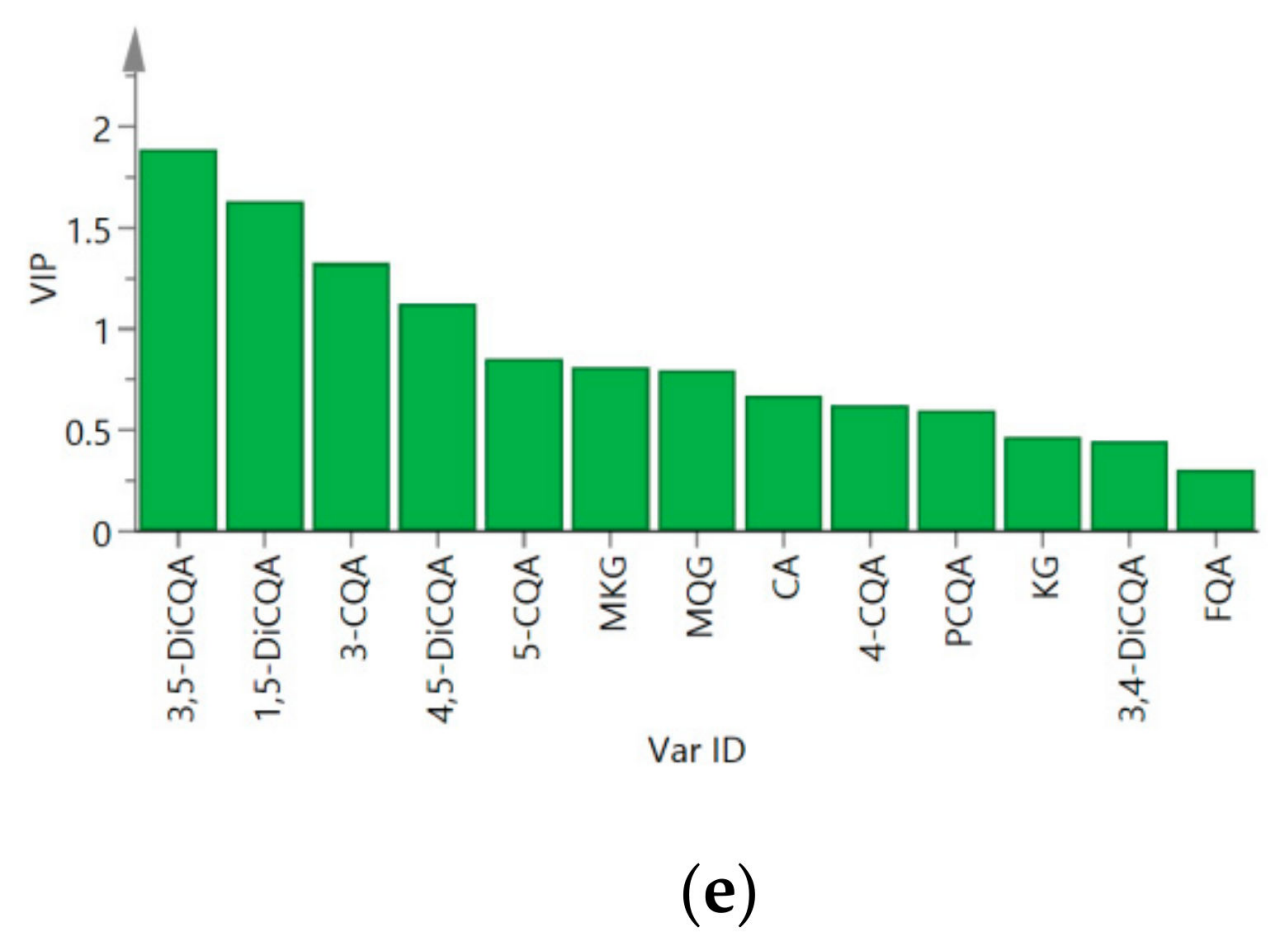
Multivariate data analysis of HPLC-MS/MS data (n = 3) of *H. tuberosus* leaves. (**a**), Score scatter plot of PCA; (**b**), Loading scatter plot of PCA; (**c**), Score scatter plot of OPLS-DA; (**d**), Loading scatter plot of OPLS-DA (X - compounds, Y - groups); (**e**), VIP was obtained from OPLS-DA. 1, CEs of different extraction methods; 2, CEs of fractions with different polarities; 3, CEs of different cultivars; 4, CEs of different growth stages of *H. tuberosus* leaves.

A total of four groups of *H. tuberosus* leaves samples were examined, including CEs obtained by different methods (group 1), fractions with different polarities (group 2), various cultivars (group 3), and different growth stages (group 4). Multivariate data analysis was performed based on the relative peak areas obtained for these four groups. PCA is a variable reduction procedure to develop a smaller number of artificial variables (called principal components, PCs) that account for most of the variations in the observed variables. The PCs could be presented graphically as a score plot (Figure 2a). In the present study, the first and second components of PCA explained 30.8% and 20.9% of variance, respectively. In terms of the identified metabolites, a loading plot of PCA (Figure 2b) showed that 3-CQA, caffeoylquinic acid isomer (4-CQA), and feruloyl-quinic acid (FQA) were more enriched in the NB fraction, while 1,5-DiCQA and 4,5-dicaffeoylquinic acid (4,5-DiCQA) were more abundant in the wild-type cultivar. Furthermore, the clustering behavior of group 4 could be attributed to caffeoylquinic acid isomer (5-CQA), CA, kaempferol-3-o-glucoside (KG), and methoxy-kaempferol glucoside (MKG). The distribution and trend of the score and loading plots of PCA presented consistency and comparability [12]. Although the two PCs resolved the measured phenolics profiles of the samples in different cultivars (negative PC1 values), PCA could not distinguish the other three groups. Thus, OPLS-DA was employed, which provided clearer separation based on PCA model [14]. In the present study, similar to the PCA, OPLS-DA scores plots presented more obvious classification of the four groups. VIP is the weighted sum of squares of the OPLS weight, and VIP of >1 is used as a criterion to identify the variables that are important to the model [15]. As shown in Figure 2c, good separation in the score plot of PC1 (24.2% of total variance) and PC2 (14.9% of total variance) of OPLS-DA was obtained. The OPLS score plot explained 79.3% of total variance (R^2^ = 0.793) with prediction goodness parameter Q^2^ = 0.699. As indicated in Figure 2d, p-coumaroyl-quinic acid might be the major component in group 1, 4-CQA might be the characteristic variable in group 2, 1,5-DiCQA might be the characteristic variable in group 3, and KG and MKG might be the characteristic components in group 4. These results are consistent with the PCA results. Variable importance in the projection (VIP) can be defined as the weighted sum of squares of the OPLS weight, and four elements (3,5-DiCQA, 1,5-DiCQA, 3-CQA, and 4,5-DiCQA, VIP>1) were found to be significant in the discrimination model for determining phenolics (Figure 2e). 

**Figure 3:**
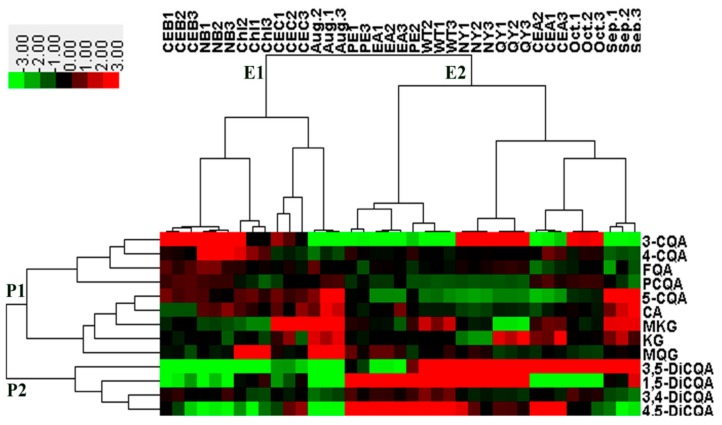
Hierarchical cluster analysis (HCA) and heatmap of bioactive phenolics and different extracts from *H. tuberosus* leaves. The colours from green to red indicate the upregulated level of compounds. The clusters E1,E2,P1,P2 and P3 refer to the different extracts samples and the phenolics classes, respectively. CE, Crude extract; A, Refluxing; B, Macerating; C, Refluxing under vacuum; PE, CE of Petroleum ether fraction; Chl, CE of Chloroform fraction; EA, CE of Ethyl acetate fraction; NB, CE of n-Butanol fraction; NY, CE of Cultivar Nan Yu; QY, CE of Cultivar Qing Yu; WT, CE of Wild type; Aug., CE of Mid-August; Sep., CE of Mid-September; Oct., CE of Mid-October.

Similar to PCA, HCA is an unsupervised algorithm that samples variables are grouped on the basis of similarities [16]. The relationships between samples and bioactive compounds are represented by the distances. The closer the distance of samples is, the more similar the samples are [17]. As displayed in Figure 3, the two classes of 13 kinds of phenolics and the two main clusters of extracts samples were obviously classified by HCA. HCA clustered extracts samples into two principal groups: cluster E1 and E2. The samples in cluster E2 were more chemically similar to each other, with similar contents of dicaffeoylquinic acid isomers. On the other hand, the samples in cluster E1 were more chemically distinct. While the bioactive phenolics were grouped into cluster P1 and P2. Cluster P2 included all the dicaffeoylquinic acid isomers (3,5-DiCQA,1,5-DiCQA, 3,4-DiCQA and 4,5-DiCQA) which were major phenolics in *H. tuberosus* leaves, while cluster P1 contained caffeoylquinic acid isomers (3-CQA,4-CQA and 5-CQA) and other minor phenolic compounds. HCA results showed that determinants such as genotype, different extraction methods, the growth location and harvesting time might affect the metabolites, which was in accordance with previous studies [17]. 

### 2.3. Antifungal Activities of Different Extracts and PLS-DA

**Figure 4:**
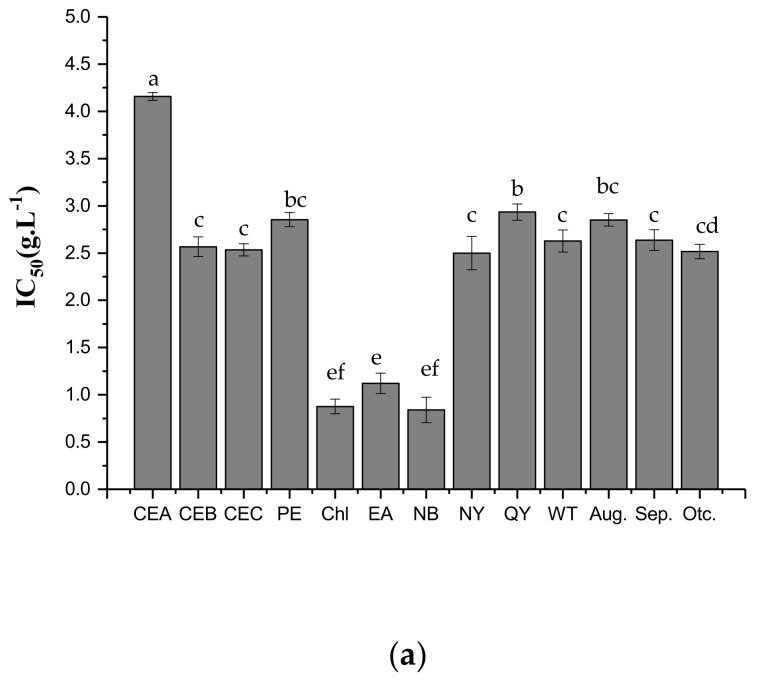

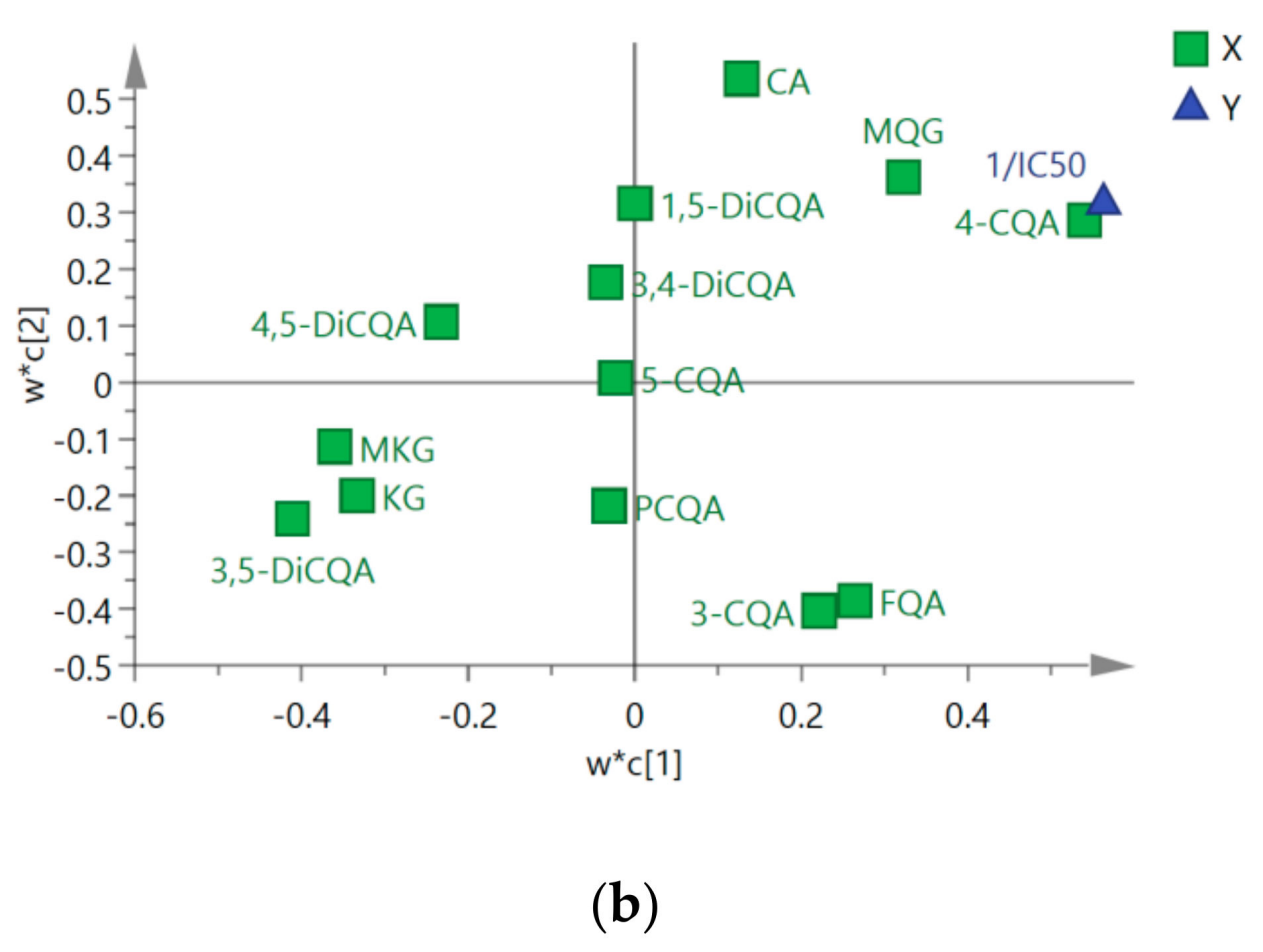
Antifungal activities of different CEs and PLS-DA of *H. tuberosus* crude extracts. (**a**), IC_50_ of different CEs of *H. tuberosus* leaves against *P. capsici* Leonian, values followed by different letters in bars are significantly different at *p* ≤ 0.05; (**b**), Loading plot of PLS-DA of *H. tuberosus* CEs based on characteristic peaks and 1/IC_50_.

The effects of different *H. tuberosus* leaves fractions on the growth of *P. capsici* Leonian were examined and represented as the concentrations producing 50% of mycelial growth inhibition (IC_50_), as reported in our previous study [4]. The *H. tuberosus* leaves CEs were potent against *P. capsici* Leonian, presenting IC_50_ of 0.839–2.853 g/L (Figure 4a). In particular, the Chl and NB fractions were more active against *P. capsici* Leonian with low IC_50_ values (0.875 and 0.839 g/L, respectively), whereas the EA fraction presented an IC_50_ value of 1.154 g/L. Furthermore, CEB and CEC were more effective with lower IC_50_ values, and cultivar Nan Yu and wild type were more potent than cultivar Qing Yu against *P. capsici* Leonian. Moreover, sample collected in mid-October at the tuber swelling stage was more potent than those collected at the flowering (mid-August) and budding (mid-September) stages. 

The reciprocal of IC_50_ (1/IC_50_) [18] could reformulate the antifungal activities of different CEs in PLS projection model, and was used as the Y variable. The higher the value of the 1/IC_50_, the higher the antifungal activity. The loading plot of PLS-DA is illustrated in Figure 4b, with Y (indicating antifungal activity) located in the upper left part of the loading plot, where most of the representative marker metabolites responsible for the antifungal activity of *H. tuberosus* leaves against *P. capsici* Leonian could be found. Among these metabolites, the most active components were 4-CQA, MQG, and CA, which were the closest to Y. 4-CQA, MQG, and CA might be the main active components in *H. tuberosus* leaves against *P. capsici* Leonian. These results are consistent with those reported in previous studies [19,20,21]. 

### 2.4. Scanning Electron Microscopy 

**Figure 5:**
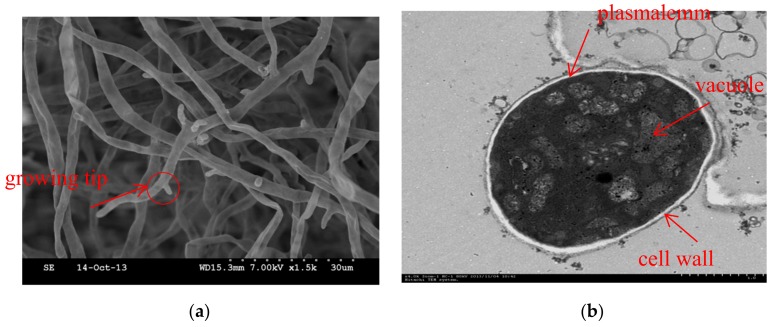

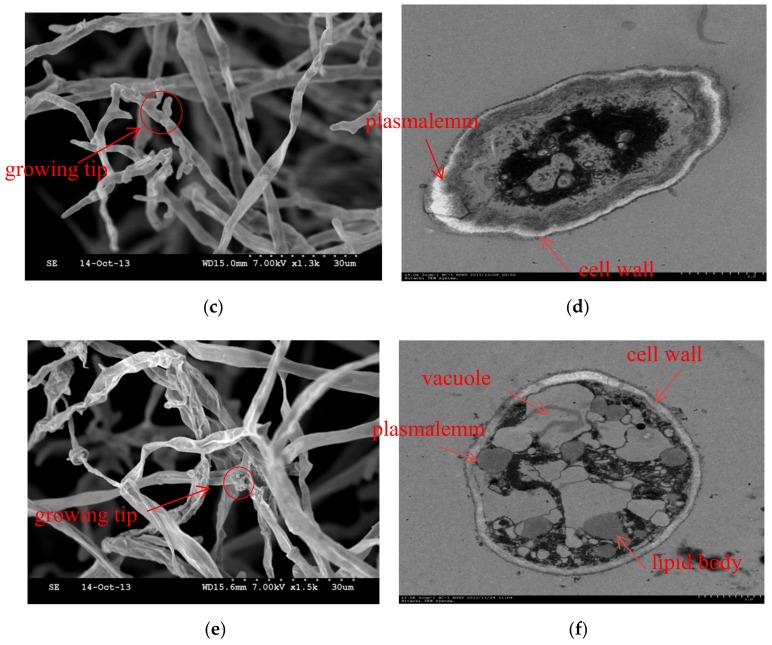
SEM and TEM images of *P. capsici* hyphae treated with different fractions (**a**,**b**), Untreated control; (**c**,**d**), Treatment with Chl fraction; (**e**,**f**), Treatment with n-butanol (NB) fraction. The Chl and NB fractions were applied at a concentration of 0.875 and 0.839 g/L (IC_50_), respectively.

Figure 5a,c,e show the scanning electron microscopy (SEM) images of mycelial growth of *P. capsici* Leonian in the control and groups treated with *H. tuberosus* crude extracts (Chl and NB fractions). At IC_50_ of the two fractions, morphological alterations (both shape and size) were noted in the treated hyphae, when compared with the control, confirming the antifungal activity of *H. tuberosus* leaves against *P. capsici* Leonian. A series of marked structural and morphological alterations in the hyphae were observed following *H. tuberosus* treatment; most of the *P. capsici* hyphae were severely damaged or collapsed, even necrotic, presenting abnormal curling, swelling, local denting, and excessive branching. Besides, some broken mycelia, or cracks on the surface of hyphae, were also observed, which indicated cell wall damage, and the growing tips of the treated hyphae were abnormal, when compared with those noted in the control. 

### 2.5. Transmission Electron Microscopy 

**Figure A1:**
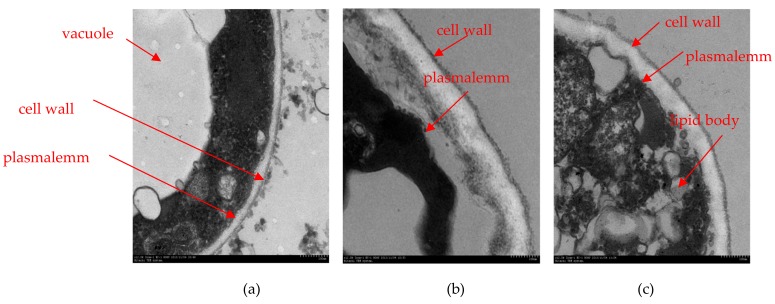
TEM images of *P. capsici* hyphae treated with different fractions. (**a**) Untreated control; (**b**) Treatment with Chl fraction; (**c**) Treatment with NB fraction.The Chl and NB fractions were applied at a concentration of 0.875 and 0.839 g/L (IC_50_), respectively.

Transmission electron microscopy (TEM) results indicated that the cellular ultrastructure of the *H. tuberosus*-treated hyphae was damaged (Figure 5b,d,f). And the figures of transmission electron microscopy at 200nm had also been listed in Appendix A (Figure A1). The cell wall of the treated *P. capsici* was thickened and plasmolysis was evident. Furthermore, Chl fraction treatment degraded the organelles of the fungus, NB fraction treatment led to the increase in lipid bodys and serious vacuolation.

## 3. Discussion

Chemometrics is an interdisciplinary research field involving multivariate statistics, mathematical modelling, and computing, and is particularly applied to understand chemical data. It has been employed for quality control of dairy products [22] as well as for determining the antioxidant, antitumor [23], antidiabetic [24], and antibacterial properties [12,25] of bioactive compounds in a sample. PCA is a very important tool, especially in the preliminary steps of multivariate analysis, for performing exploratory investigation to obtain an overview of data and determine patterns in complex experimental data. Moreover, PCA helps in identifying the difference among the samples as well as the variables that contribute most to this difference.

In order to classify the samples on the basis of the bioactive compounds, an HCA dendrogram with heat-map is performed to not only analyse and visualize the similarity between different samples but also to clarify the difference in compounds among various samples [26].

It must be noted that PLS-DA allows classification of samples based on their bioactivities, and could be used to identify components predominantly involved in bioactivities [27]. The PLS-based approach is a robust regression technique used to investigate the relationships between two blocks of data, usually denoted as X- and Y-block, and has predictive applications. In this model, bioactivities might be considered as Y-block, and PLS-DA could be applied to classify the samples based on their bioactivities, as well as identify the components that are predominantly involved in bioactivities [27]. 

Phenolic acids such as CA, chlorogenic, ferulic, and p-coumaric acids [28,29] have been noted to be active against many human pathogens, including Staphylococcus aureus, etc. Besides, CA and chlorogenic acid have also been used to inhibit zoonotic pathogens such as Listeria monocytogenes [30]. In addition, some phenolic acids, including CA, chlorogenic, isochlorogenic, ferulic, and p-coumaric acids, have also been found to inhibit Sclerotinia sclerotiorum, *P. capsici*, and other phytopathogenic fungi [19,20,21]. Although these compounds can be employed either individually or in combination to effectively inhibit phytopathogenic fungi [31], their combined usage might be more effective against *P. capsici*, when compared with their individual application. In our previous studies, crude extracts (CEs) of *H. tuberosus* leaves possess antifungal activities against phytopathogenic fungi such as *P. capsici* Leonian and Rhizoctonia cerealis [4]. In the present study, SEM and TEM evaluation revealed the mechanism of Chl and NB fractions of *H. tuberosus* leaves against *P. capsici* Leonian. The effect on the morphology and ultrastructure of pathogenic fungi can visually characterize the antifungal activity, which is consistent with our antifungal activity tests. SEM and TEM showed that the antifungal effects of Chl fraction and NB fraction were different. A series of marked alterations in *P. capsici* hyphae with Chl fraction treatment were observed, such as the growing tips increasing sharply, the hyphae denting locally, and even the fractures occurred by SEM. Furthermore, aberrant morphogenesis was also manifested with Chl fraction treatment as cell wall thickening and plasmolysis, even organelle degrading by TEM. The noteworthy alterations with NB fraction treatment were swells, cracks of hyphae and the degradation of growing tips by SEM.TEM showed the development of a thicker cell wall, plasmolysis, the increase in lipid bodies and serious vacuolation. Vacuole also plays an important role in maintaining the fungal turgor pressure, and it is more important for mycelial growth [32]. The fungal cell wall as the target of antifungal active ingredients is a unique organelle which function in cell growth and normal physiological activities. The main functions of it are provided for the shape of the cell resistance to the difference in internal and external osmotic pressure, selective permeability to large molecules and accumulation of molecules important to the physiology of the cell, such as nutrition and metabolites [33]. It’s probably because that the active compounds such as phenolics could inhibit the synthesis of cell wall either directly or indirectly from destroying the cell integrity and acting on the cell membrane with leakage of cytoplasm to interfere with the normal physiological metabolism of the hyphae [34]. The phenolics might have contributed to restrict the invasion of *P. capsici* [35,36], which was consistent with our research. Phenolics are secondary metabolites as signal compounds, pigments, internal physiological regulators or chemical messengers, which might play a dominant role in the resistance mechanism of plants against pathogens [37]. These findings could facilitate further investigation on the inhibitory mechanism of phenolic acids, applied either individually or in combination, against *P. capsici* Leonian. 

## 4. Materials and Methods 

### 4.1. Chemicals and Plants 

Gallic acid was obtained from Sinopharm Chemical Reagent Co., Ltd. (Shanghai, China), and 3-CQA and rutin were bought from Aladdin Reagent Co., Ltd. (Shanghai, China). Other standard samples were purchased from Yuanye Biological Technology Co., Ltd. (Shanghai, China). All other analytical-grade chemicals were obtained from Shoude Experimental Equipment Co., Ltd. (Nanjing, China). 

CEs of different extraction methods and CEs of fractions with different polarities were the extracts from the leaves of *H. tuberosus* cultivar Nan Yu collected at the end of October 2010. The leaves of three *H. tuberosus* cultivars [wild type, Nan Yu, and Qing Yu] were collected at maturity from Dafeng District (Jiangsu, China) at the end of October 2011e leaves of cultivar Nan Yu were collected from mid-August to mid-October 2012 at different growth stages, including budding, flowering, and tuber swelling stages, respectively. All the collected leaves were air-dried at room temperature.

### 4.2. Preparation of Extracts

The dried and crushed leaves (1 kg) were extracted by three different methods, including refluxing(A) for 3 h, macerating(B) overnight, and refluxing under vacuum(C)for 3 h with 70% *v/v* ethanol (EtOH) for three times. Then, the crude extract of refluxing under vacuum (CEC) was chosen for following research. After evaporation under reduced pressure, 120.85 g of dried EtOH CE were suspended in 500 mL of water and partitioned sequentially with Petroleum ether (3 × 500 mL), Chloroform (3 × 500 mL), Ethyl acetate (3 × 500 mL), and n-Butanol saturated with water (3 × 500 mL). After removing the solvents, four fractions were obtained. The yields of PE, Chl, EA, NB, and CE of water fraction were 5.25, 13.50, 20.42, 24.99, and 55.08 g, respectively. After evaporation under reduced pressure, the dry residues were re-dissolved in 25 mL of methanol and used for colorimetric and chromatographic analyses, while the other residues were stored in air-tight bottles at 4 °C for antifungal assays. For HPLC-MS/MS analysis, all the samples were filtered through a 0.22-μm cellulose acetate filter (Millipore Corp., Bedford, MA, USA) before injections.

### 4.3. HPLC-MS/MS Analysis

HPLC-MS/MS analysis of phenolics in *H. tuberosus* CEs was performed using an Agilent 1200 series HPLC system (Agilent Technology Co. Ltd., Palo Alto, CA, USA), composed of a diode array detector and an Agilent 6400 series triple quadrupole mass spectrometer equipped with an electrospray ionization (ESI) source. The data were collected and processed via a personal computer running Agilent MassHunter workstation (Micromass, Qualitative Analysis Version B.01.03 of Agilent Technology Co. Ltd., Palo Alto, CA, USA). A reverse-phase Eclipse XDB-C18 column (250 mm × 4.6 mm, 5 µm, Agilent Technology Co. Ltd., Palo Alto, CA, USA) was used for separation. The mobile phases consisted of methanol (A) and 1.0% *v/v* formic acid aqueous solution (B). Gradient elution was started with 30% of A and ascended to 50% of A in 45 min. The flow rate was kept at 0.8 mL/min, and the column temperature was 30 °C. The samples were filtered through a 0.22-µm filter prior to HPLC injection, and the injection volume was 5 µL. UV-Vis absorption spectra were recorded online from 200 to 600 nm during HPLC analysis, and phenolics were detected at the wavelength of 330 nm. 

Mass and MS/MS spectra were achieved by ESI in negative modes. The voltages used were 4000 V for the source capillary and 10 V for the extraction cone: source temperature was 150 °C and desolvation temperature was 350 C. The electrospray probe-flow was adjusted to 70 mL/min. The ESI-MS and ESI-MS/MS spectra were obtained by scanning from 200 to 1200 *m*/*z*. The MS/MS fragmentations were conducted with 10%–50% energy. 

### 4.4. Measurement of TPC and TFC

TPC was determined using the Folin–Ciocalteu reagent with gallic acid as the standard [38,39]. Each CE (0.5 mL of 400 g/L) was mixed with 0.5 mL of the Folin–Ciocalteu reagent freshly prepared in our laboratory, 2.0 mL of 10% *w/v* sodium carbonate solution, and 3.0 mL of distilled water. After 2 h of reaction under ambient temperature in dark, the absorbance was measured at 760 nm. A calibration curve with equation: y = 0.0029x + 0.0107 (R^2^ = 0.9991) was constructed using gallic acid solutions in the range of 1.470–294 mg/L. The results were expressed in mg gallic acid equivalents/g of dried sample.

TFC was evaluated by aluminum chloride (AlCl_3_) colorimetric assay [40,41,42]. Briefly, 1 mL of 5% AlCl_3_ in methanol was mixed with the same volume of extracts (400 g/L). Then, the mixture was vortexed and absorbance was measured after 15 min at 430 nm. The TFC was expressed in mg/g of rutin equivalent (RE). A calibration curve was constructed by preparing rutin solutions at concentrations of 5.36–346 mg/L using equation: y = 0.0013x + 0.0023 (R^2^ = 0.9995).

### 4.5. Microorganism 

The plant pathogen *P. capsici* Leonian, donated by Jiangsu Academy of Agricultural Sciences, was incubated on potato dextrose agar (PDA) at 23 °C in dark.

### 4.6. Antifungal Assay 

The antifungal activities of the CEs and fractions of *H. tuberosus* leaves against *P. capsici* Leonian were evaluated by growth inhibition bioassay [4]. The tested reagents were dissolved in acetone and added to sterile culture medium (PDA) at specific concentrations. Following thorough mixing, the medium was poured into Petri dishes (9 cm i.d.). The presence of small amount of acetone along with each reagent had no effect on pathogen growth. The 4 × 4-mm agar plugs infected with the fungus were incubated on agar plates (1 plug/plate) at 23 °C in dark. The colony growth diameters were measured for 7 days. All treatments were performed in quadruplicate, and plates without any additives were used as control. The IC_50_ values, mean values, standard deviations, and P-values were evaluated using SPSS software version 20.0 for windows (SPSS lnc., Chicago, IL, USA).

### 4.7. SEM

*P. capsici* Leonian mycelium cells and mycelial plugs collected from the rim of the fungal colonies of the same age (23 °C for 7 days) both in the control and treatment groups (Chl and NB fractions at IC_50_) were examined under SEM. In brief, the samples were fixed with 3% glutaraldehyde for 4 h at room temperature, washed with phosphate buffer (pH of 6.8) for six times, dehydrated stepwise with graded aqueous series of acetone (25%, 50%, 75%, and 100%, 10 min/step), critical-point-dried with CO_2_ for 2 h, and mounted onto aluminum stubs using double-sided adhesive tape. Finally, the samples were sputter-coated with 20 nm of gold palladium for subsequent observation of morphological changes using a Hitachi S-3000N scanning electron microscope (Tokyo, Japan).

### 4.8. TEM

The mycelial plugs were collected as mentioned earlier, and the samples were fixed with 3% glutaraldehyde for 4 h and then rinsed with phosphate buffer. Then, the samples were post-fixed with OsO_4_ (1%) at 0 °C for 2.5 h and dehydrated in a graded series of EtOH and embedded in Epon/Araldite resin. Subsequently, ultrathin sections were stained with uranyl acetate and lead citrate and observed using a Hitachi H-7650 transmission electron microscope (Tokyo, Japan).

### 4.9. Statistical Analysis

TPC, TFC, and phenolics compounds content in different fractions of *H. tuberosus* leaves were reported as mean ± SD from triplicate results. A semi-quantitative analysis based on relative peak areas was conducted to evaluate the relative contents of phenolics in different fractions of *H. tuberosus* leaves. Statistical analysis was performed using analysis of variance (ANOVA), with statistical significance at *p* ≤ 0.05. PCA and OPLS-DA were performed to understand the correlation between the changes in TPC. PLS-DA was used to analyze the relationship between phenolics and their antifungal activities, and 1/IC_50_ values could reformulate their antifungal activities. The obtained results were processed by using SIMCA-P Version 14.1 (Umetrics, Umeå, Sweden). An HCA (Hierarchical Cluster Analysis) dendrogram was used to construct heat-map and cluster analysis by Cluster 3.0 and Java TreeView Software (Stanford University School of Medicine, Stanford, CA, USA).

## 5. Conclusions

In this study, the antifungal activities of different extracts and compounds from *H. tuberosus* leaves against *P. capsici* Leonian were evaluated through chemometric analyses, including HPLC-MS/MS and multivariate data analysis. PCA, OPLS-DA and HCA were applied to distinguish the four groups of *H. tuberosus* leaves samples, including three CEs obtained by different methods (CEA, CEB, and CEC), four fractions (PE, Chl, EA, and NB fractions), three *H. tuberosus* cultivars, and three growth stages of cultivar Nan Yu. The TPC could be categorized based on 3,5-DiCQA, 1,5-DiCQA, 3-CQA, and 4,5-DiCQA levels, which were predominant in all the samples. The antifungal activity assay revealed that Chl and NB fractions were more active against *P. capsici* Leonian with lower IC_50_ values, and PLS-DA indicated that 4-CQA, MQG, and CA might be the main active components in *H. tuberosus* leaves against *P. capsici* Leonian. Furthermore, SEM and TEM evaluation demonstrated structural deformities in *P. capsici* Leonian treated with Chl and NB fractions, suggesting the antifungal effects of *H. tuberosus* leaves against this phytopathogenic fungus. These results imply that *H. tuberosus* leaves of Chl and NB fractions from cultivar Nan Yu with high concentration of phenolics might be a promising source of natural fungicides.

## Figures and Tables

**Table 1:** Relative percentage of main phenolic acids in *H. tuberosus* leaves extracts.

Sample ^1^	5-CQA	3-CQA	4-CQA	CA	PCQA	FQA	3,4-DiCQA	3,5-DiCQA	1,5-DiCQA	MQG	4,5-DiCQA	MKG	KG
CEA	0.49 ± 0.06 g	14.14 ± 0.12 g	2.51 ± 0.43 bc	1.26 ± 0.19 def	2.52 ± 0.41 a	1.11 ± 0.16 de	2.96 ± 0.53 bc	23.76 ± 0.19 c	3.43 ± 0.19 j	1.54 ± 0.23 de	17.08 ± 0.65 b	5.27 ± 0.16 c	4.19 ± 0.67 a
CEB	3.20 ± 0.24 c	25.64 ± 0.72 c	1.89 ± 0.09 de	0.48 ± 0.11 f	2.53 ± 0.23 a	2.49 ± 0.27 ab	3.18 ± 0.17 abc	6.42 ± 0.37 h	8.37 ± 0.75 g	1.13 ± 0.25 de	11.14 ± 1.14 e	4.03 ± 0.35 e	2.22 ± 0.24 bc
CEC	3.06 ± 0.26 cd	17.46 ± 0.77 f	1.08 ± 0.10 gh	2.17 ± 0.78 bc	1.47 ± 0.42 bc	1.68 ± 0.30 cde	2.73 ± 0.20 cd	9.70 ± 0.34 g	10.04 ± 1.03 f	1.54 ± 0.33 de	12.73 ± 1.23 d	7.10 ± 0.24 b	2.62 ± 1.27 b
PE	2.30 ± 0.25 e	13.94 ± 0.47 g	2.12 ± 0.09 cd	1.44 ± 0.10 cde	1.29 ± 0.50 cd	1.36 ± 0.31 cde	3.54 ± 0.14 a	11.96 ± 0.87 f	14.63 ± 0.90 d	1.88 ± 0.79 cd	17.62 ± 0.49 b	4.71 ± 0.17 d	2.29 ± 0.09 bc
Chl	2.65 ± 0.28 de	17.28 ± 1.39 f	2.76 ± 0.47 b	1.46 ± 0.12 cde	0.70 ± 0.21 de	1.61 ± 0.21 cde	1.94 ± 0.21 ef	7.42 ± 0.18 h	10.62 ± 0.69 ef	6.57 ± 0.13 a	9.63 ± 1.15 f	2.33 ± 0.12 g	1.62 ± 0.28 c
EA	0.67 ± 0.02 g	11.32 ± 0.51 i	1.53 ± 0.13 efg	1.93 ± 0.61 bcd	1.33 ± 0.35 cd	1.86 ± 0.18 bcd	3.34 ± 0.18 ab	8.73 ± 0.30 g	19.20 ± 0.50 c	2.41 ± 0.30 c	15.57 ± 0.18 c	3.65 ± 0.50 ef	2.37 ± 0.05 bc
NB	2.50 ± 0.29 e	30.64 ± 1.03 a	4.71 ± 0.31 a	2.38 ± 0.51 b	2.26 ± 0.18 a	2.65 ± 0.22 a	2.96 ± 0.59 bc	4.06 ± 0.14 j	7.24 ± 1.39 hi	1.26 ± 0.25 de	8.59 ± 0.53 f	3.22 ± 0.14 f	1.91 ± 0.42 bc
NY	0.25 ± 0.04 g	27.79 ± 0.52 b	1.54 ± 0.07 efg	0.48 ± 0.06 f	0.59 ± 0.04 e	1.82 ± 0.07 bcd	2.40 ± 0.06 de	26.44 ± 1.18 b	14.18 ± 0.37 d	1.69 ± 0.35 cde	13.12 ± 1.12 d	3.75 ± 0.13 e	0.56 ± 0.16 d
QY	0.65 ± 0.04 g	24.08 ± 0.46 d	1.53 ± 0.07 efg	0.90 ± 0.06 ef	0.62 ± 0.02 e	2.10 ± 0.02 abc	2.27 ± 0.04 de	23.76 ± 0.36 c	20.58 ± 0.50 b	0.89 ± 0.20 e	12.71 ± 0.22 d	1.34 ± 0.04 h	4.55 ± 0.46 a
WT	0.63 ± 0.21 g	11.54 ± 0.42 hi	1.29 ± 0.22 fg	0.86 ± 0.06 ef	0.54 ± 0.02 e	1.41 ± 0.19 cde	3.70 ± 0.25 a	17.48 ± 0.96 e	23.41 ± 0.72 a	1.55 ± 0.25 de	19.30 ± 0.71 a	5.72 ± 0.44 c	2.15 ± 0.04 bc
Aug.	4.5 ± 0.64 b	7.15 ± 0.80 j	0.57 ± 0.29 i	3.16 ± 0.52 a	2.07 ± 0.11 ab	1.94 ± 0.87 abc	1.72 ± 0.08 f	5.39 ± 0.23 i	2.45 ± 0.44 j	4.88 ± 1.10 b	5.00 ± 0.47 g	7.62 ± 0.40 a	4.35 ± 0.50 a
Sep.	7.33 ± 0.29 a	12.67 ± 1.03 h	0.80 ± 0.17 hi	3.88 ± 0.97 a	1.25 ± 0.87 cd	1.01 ± 0.86 e	2.01 ± 0.48 ef	18.52 ± 1.05 d	11.85 ± 1.20 e	1.86 ± 0.23 cd	9.67 ± 1.43 f	6.76 ± 0.16 b	3.85 ± 0.71 a
Oct.	1.70 ± 0.22 f	19.55 ± 0.50 e	1.70 ± 0.52 def	1.34 ± 0.17 de	2.23 ± 0.16 a	1.51 ± 0.30 cde	3.31 ± 0.17 ab	33.56 ± 0.30 a	6.80 ± 0.08 i	0.84 ± 0.01 e	11.74 ± 0.58 de	3.81 ± 0.14 e	2.37 ± 0.15 bc

Values are expressed as mean ± SD of triplicate measurements. Values followed by different letters in columns are significantly different at *p* ≤ 0.05; ^1^ CE, Crude extract; A, Refluxing; B, Macerating; C, Refluxing under vacuum; PE, CE of Petroleum ether fraction; Chl, CE of Chloroform fraction; EA, CE of Ethyl acetate fraction; NB, CE of *n*-Butanol fraction; NY, CE of Cultivar Nan Yu; QY, CE of Cultivar Qing Yu; WT, CE of Wild type; Aug., CE of Mid-August; Sep., CE of Mid-September; Oct., CE of Mid-October.

## Appendix A

Here are the figures of transmission electron microscopy at 200 nm (Figure A1). The changes might be necrotic, which were the same as the figures of Transmission electron microscopy at 1um (Figure 5). It’s probably because that the active compounds could inhibit the synthesis of cell wall either directly or indirectly from destroying the cell integrity and acting on the cell membrane with leakage of cytoplasm to interfere with the normal physiological metabolism of the hyphae.
